# Simultaneous determination of serotonin and its precursor tryptophan in small volumes of human serum using HPLC-ECD

**DOI:** 10.1016/j.mex.2026.104190

**Published:** 2026-10-02

**Authors:** Dorothee Licht, Alexander Duscha, Tobias Hegelmaier, Christiane Desel, Aiden Haghikia

**Affiliations:** a Department of Neurology, Otto-von-Guericke University, Leipziger Straße 44, Magdeburg, 39120, Germany; b Department of Neurology and Clinical Neurophysiology, Hannover Medical School, Carl-Neuberg-Straße 1, Hannover, 30625, Germany

**Keywords:** Electrochemical detection, Serum metabolite measurement, Chromatography, Neurotransmitter

## Abstract

Serotonin (5-HT) is not only a key neurotransmitter in the brain but also plays an important role in peripheral tissues e.g. in the gastrointestinal (GI) tract. The multitude of processes in which 5-HT is involved explains why recent studies have linked altered 5-HT levels to Long Covid (LC) and fatigue. LC is a post-viral syndrome which can occur after a SARS-CoV-2 infection with various symptoms, amongst others including fatigue. We present an optimized bioanalytical HPLC method with electrochemical detection (ECD) for the simultaneous detection of 5-HT and its precursor tryptophan (W) in small volumes of human serum. We validated the method with a calibration curve better representing concentrations expected in human serum; with the possibility to concurrently measure W and low 5-HT concentrations. Sample comparability and standardization were improved through an adapted sample preparation, reducing transfer errors and increasing recovery of the internal standard (IS). The simple preparation involved only two steps: protein precipitation and sample dilution. After a brief series of validation measurements of the optimized method, it was applied to human serum from an observational study cohort.

• Easy-to-use method for detecting 5-HT and W in human serum without the need for expensive buffer ingredients or pre-filter units.
• Enables measurement of triplicates with less than 100 µL serum.

## Graphical abstract

**Figure:**
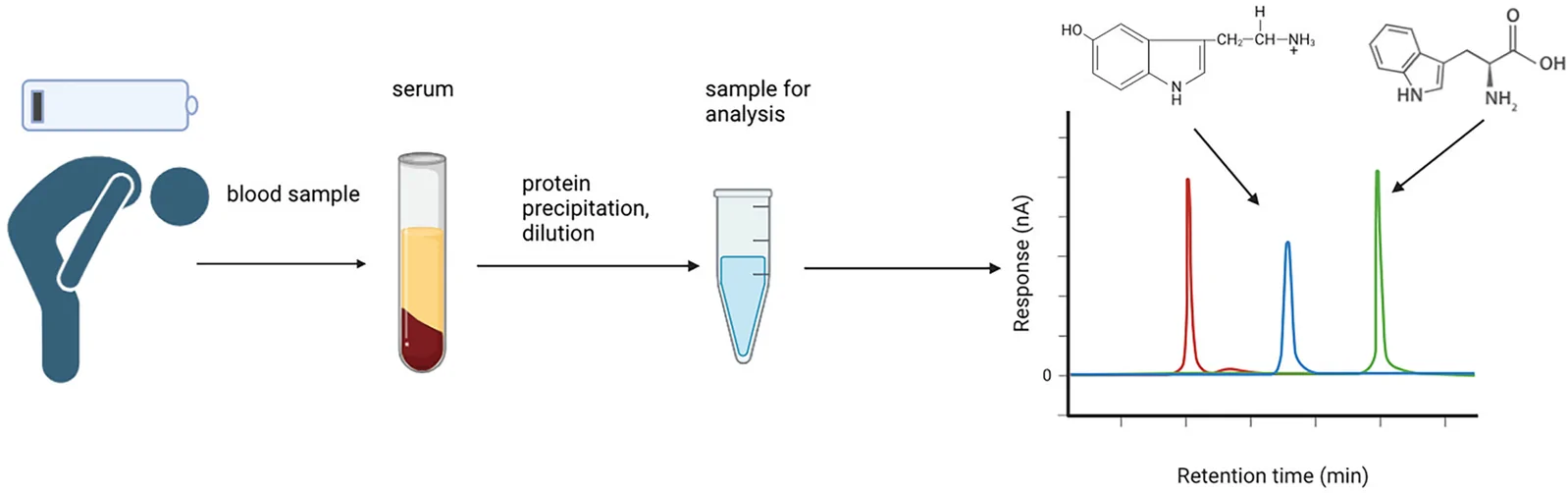

## Specifications table

**Table:** 

**Subject area**	Biochemistry, Genetics and Molecular Biology
**More specific subject area**	*Analytical chemistry*
**Name of your method**	*Chromatographic analysis of serotonin and tryptophan in human serum*
**Name and reference of original method**	*A. Gallegos, R.R. Isseroff, Simultaneous determination of tryptophan, 5-hydroxytryptophan, tryptamine, serotonin, and 5-HIAA in small volumes of mouse serum using UHPLC-ED, MethodsX, v9, 2022.* https://doi.org/10.1016/j.mex.2022.101624.
**Resource availability**	Reagents: 5-Hydroxy-Nω-Methyltryptamine oxalate 99% (H45132-100MG) from Sigma-Aldrich, Germany; Acetonitrile, ≥99.9%, gradient grade, suitable for HPLC (34851-1L) from Sigma-Aldrich, Germany; Citric acid monohydrate, ≥99.5% (5110.1) from Carl Roth, Germany; DL-Tryptophan ≥98% (T3300-5G) from Sigma-Aldrich, Germany; EDTA disodium salt dehydrate 99.0-101.0% (E5134-100G) from Sigma-Aldrich, Germany; Formic acid ACS reagent, ≥98% (33015-1L-M) from Sigma-Aldrich, Germany; Methanol, suitable for HPLC 99.9% (34860-2.5L-R) from Sigma-Aldrich, Germany; Perchloric acid ACS reagent 70% (244252-500ML) from Sigma-Aldrich, Germany; Serotonin-hydrochloride ≥98% (H9523-25MG) from Sigma-Aldrich, Germany; Sodium hydroxide 98-100.5% (06203-1KG) from Sigma-Aldrich, Germany; Water suitable for HPLC (270733-2.5L) from Sigma-Aldrich; for first testruns: Carl Roth A511.2, both Germany. Chromatography/data analysis: Alexys Neurotransmitter Analyzer from Antec Scientific, Netherlands; Clarity CDS version 8.4 from Data Apex, Czech Republic; Kinetex LC C18 column, (150 × 2.1 mm; 2.6 µm) (00F-4462-AN) from Phenomenex, USA; Sample vials polypropylene for HPLC (4001048) from Microbiotech, Sweden; SecurityGuard ULTRA holder and cartridge from Phenomenex, USA; SenCell 2 mm GC sb (116.4120) from Antec Scientific, Netherlands; Snap cap, 8 mm Precision Fit Clear PTFE/Silicone with Slit Lined (5870-08) from Dunn Labortechnik/J.G. Finneran, USA; Whatman Aqueous In-Line filter/Degasser disposable filter (6726-5002A) from Cytiva, USA; Other (for Serum/sample/buffer preparation): 2.0 ml SC micro tube PCR-PT (72.694.406) from Sarstedt, Germany; 7.5 ml S-Monovette Z tubes (01.1601.001) from Sarstedt, Germany; Sonication bath from Elma, Germany

## Background

LC is a post-viral syndrome associated with a previous SARS-CoV-2 infection. SARS-CoV-2 was first reported in 2019 and has since then infected several million people worldwide, of whom around 10% show symptoms of LC [1,2]. These can vary between individuals and frequently include fatigue, memory loss and other neurocognitive impairments. LC can also have impacts on the GI tract, lungs, heart and other organs. The molecular etiology of LC remains unclear [3]. One possible theory being discussed in the context of fatigue but also LC is 5-HT deficiency [[3], [4], [5], [6], [7]]. 5-HT is not only a neurotransmitter in the brain but is also involved in processes in peripheral tissues, e.g. during inflammation [8]. The reason why 5-HT deficiency is associated with fatigue is due to the known effects of 5-HT on sleep, appetite, pain and its regulatory function in the GI tract and cardiovascular/pulmonary system [9]. Ninety-five % of 5-HT in the body is synthesized, stored and released in intestinal cells, the so-called enterochromaffin cells. 5-HT is synthesized from the amino acid W by the enzyme tryptophan hydroxylase [10]. Therefore, the comparative measurement of W and 5-HT in the serum of different groups of test subjects is highly desirable. One method that has already been used for decades for measuring 5-HT and its precursor W in blood samples is HPLC [11]. ECD is considered to be particularly sensitive [12]. Existing methods often work with ion-pairing agents such as 1-octanesulfonic acid, special filter units for pre-treatment [13], are focussed on the measurement of 5-HT from (rat) brain tissue or cerebrospinal fluid [14,15] or use different detectors for the quantification of 5-HT and W [16]. In this protocol, we work without ion-pairing agents and without special filter units in order to enable a more cost-effective analysis. The focus is on accurate measurement of 5-HT and W in small volumes of human serum in contrast to the original method [17], which focused on mouse serum. Additionally, the method was optimized with regard to repeatability and sample comparability by a modified sample precipitation process and an improved recovery of the IS for standardization. The usage of a lower potential of the electrochemical flow cell in contrast to the original method [17] improved the equilibration time and shortened the daily device preparation.

## Method details

### Apparatus and HPLC conditions

For the separation of the samples a Kinetex LC C18 column, 2.1 mm x 150 mm, 2.6 µm particle size was used. The ALEXSYS Neurotransmitter analyzer consisted of an OR 110 degasser, an LC 110 S pump, an AS 110 automatic sample introduction system and the DECADE Elite electrochemical detector. For the detection, the system was equipped with a SenCell flow cell with a 2 mm glassy carbon working electrode. A 96:4 (v/v) mixture of 50 mM citrate buffer:acetonitrile was used as mobile phase and ran at 100 µL/min; its pH was adjusted to 4.3 with 50% NaOH. For filtration of the mobile phase a Whatman Aqueous IFD disposable filter was installed between the mobile phase reservoir and the HPLC pump. The temperature for the column and detection was set at 37°C and the detection potential was 0.9 V against an Ag/AgCl reference electrode. The noise filter was set to 0.5 Hz while the range was 500 nA. The injection was performed in partial-loop mode with 2 µL injection volume. For the measurements polypropylene HPLC sample vials were used in a 96-well plate and placed in the automatic sample introduction system at 4°C. The final run time per sample was 13.5 min.

### Mobile phase

To ∼900 mL of distilled water in a clean graduated glass cylinder 10.08 g citric acid monohydrate was added and mixed well until the citric acid monohydrate was completely dissolved and adjusted to pH 4.3 with 50% NaOH solution (∼6 mL). Solution was filled up to 960 mL with distilled water and 40 mL acetonitrile was added, mixed well and transferred into a glass bottle. Mobile phase was degassed in sonication bath for ∼15 min.

### Standard preparation

Individual stock solutions of W, 5-HT-hydrochloride and the IS compound 5-Hydroxy-Nω-Methyltryptamine oxalate (synonyme: N-methylserotoninoxalate) were prepared by adding HPLC water (for 5-HT-hydrochlorid and the IS) or a mixture of 7:3 (v/v) methanol:water (for W) to obtain 1 mM solutions. These aliquots were stored short-term (several days) at -20°C and long-term at -80°C.

At the day of analysis, these aliquots were thawed at room temperature (RT) to prepare the working solutions (WS) as a 1:10 dilution of W and 5-HT 1 mM stocks in HPLC water to be used for the standard curve. Before usage the WS of 5-HT was further diluted (see Table 1). The final standards were prepared by adding the appropriate amounts of the (pre-diluted) WS, perchloric acid, protein precipitation (PP) buffer, IS and HPLC water (see Table 2).

**Table 1:** Pre-dilution series 5-HT, WS = working solution, PD = pre-dilution.

	Pre-dilution a (PDa)	Pre-dilution b (PDb)	Pre-dilution c (PDc)
WS/pre-dilution	200 µL WS	200 µL PDa	200 µL PDb
HPLC Water	600 µL	600 µL	600 µL

**Table 2:** Preparation of standard mixture solutions (points 1 to 4); for recipe see sample preparation and preparation of protein precipitation (PP) buffer, WS = working solution, HClO_4_ = perchloric acid.

	Calibration point 1 (50 nM 5-HT, 50 nM W, 8 µM IS)	Calibration point 2 (200 nM 5-HT, 2 µM W, 8 µM IS)	Calibration point 3 (800 nM 5-HT, 5 µM W, 8 µM IS)	Calibration point 4 (3.2 µM 5-HT, 8 µM W, 8 µM IS)
PP buffer	2 mL	2 mL	2 mL	2 mL
HClO_4_ 70%	60.4 µL	60.4 µL	60.4 µL	60.4 µL
IS 1mM	80 µL	80 µL	80 µL	80 µL
W WS	5 µL	200 µL	500 µL	800 µL
5-HT	320 µL PDc	320 µL PDb	320 µL PDa	320 µL WS

Fill up to 10 mL with HPLC water

### Calibration

On the basis of previous literature N-methylserotoninoxalate was chosen as the IS [17,18]. It was used in the sample preparation but also in the standard curve, enabling the recognition of volume errors occurring throughout the different steps of precipitation and pipetting. N-methylserotoninoxalate was sufficiently separated from other metabolites, especially W, enabling the differentiation of the peaks in the integration process. The IS was added to all samples and calibration points at a concentration of 8 µM. The calibration curve consisted of four calibration points with the lowest calibration point being 50 nM for both 5-HT and W.

### Blood collection

The blood was taken as part of an observational study cohort in the University Hospital Magdeburg, Department of Neurology. Venous blood samples were withdrawn into 7.5 mL S-Monovette tubes containing clot activator. The blood was allowed to coagulate for up to an hour and afterwards the samples were centrifuged at 935 x g for 15 minutes. The sera were transferred to 2 mL micro tubes, immediately frozen and stored at -80°C until analysis. The samples were collected within approximately two years and then stored for up to two years.

### Sample preparation and preparation of protein precipitation buffer

For the PP buffer 400 µL distilled water and 10 µL formic acid were added to a 10 mL measuring cylinder and the volume was filled up to 10 mL with methanol which results in a final concentration of 0.1% (v/v) formic acid in the PP buffer.

Thawed serum samples were gently vortexed and 20 µL were transferred into a 1.5 mL tube, then 40 µL of PP buffer was added and mixed thoroughly. This mixture was allowed to sit for 1′, then vortexed for 30 seconds at full speed and centrifuged at 21.000 x g for 10 minutes at RT. 30 µL of the supernatants were transferred to HPLC sample vials and mixed with 70 µL of 0.1N perchloric acid/2 mM EDTA containing also the IS (11.429 µM to reach a final concentration of 8 µM IS in 100 µL sample volume). This results in a final dilution of the samples of 1 to 10. Processed samples were usually measured within 12 hours while being constantly cooled at 4°C in the cooling unit.

### HPLC Adjustment

The main point of the method optimization was to find the conditions for a given HPLC setting which would allow us to have the best fit of equilibration time, peak height/area, noise, retention time, repeatability and integration. The first step was the evaluation of different potential levels from 0.8-1.0 V. For 5-HT lower potentials than 1.0 V are also possible without a huge change in the peak area [19] but the analysis of W requires a higher working potential [20]. A higher potential requires a longer equilibration time of the SenCell. The second step was the evaluation of the effect of an adjustment of the cell working volume of the SenCell (spacing set to position 1 and 2) especially with regard to the effect on the peak size and integration. The next step was the improvement of the sample preparation process to increase the repeatability. This included increasing the serum sample volume from 10 to 20 µL (with the corresponding volume of PP buffer) and the direct addition of IS to perchloric acid for samples or in the standard mix respectively for higher recovery (instead of addition into the PP buffer). Last, we optimized the calibration curve in order to better represent the range of 5-HT concentrations found in serum of healthy humans, which improved the detection of 5-HT in the serum samples. This also included a pre-dilution series for 5-HT to reduce the number of small-volume transfers and increase repeatability. The possible effect of an increase of the flow rate on the peak shape, retention time and resolution was tested with a serum sample. However, this resulted only in a reduced run time but did not improve peak resolution. For this reason, effects of the flow rate were not investigated further.

## Method validation

The specificity was evaluated by analyzing not only the main ingredient of the PP buffer methanol and additionally perchloric acid/EDTA (part of the storage solution used for dilution) but also the mobile phase, the standards, the IS and a processed serum sample respectively. No peaks were detectable in the blank sample (mobile phase) at the retention time of the analytes as seen in Fig. 1 and the method is specific enough to allow the simultaneous separation of the two analytes and the IS from each other and from the buffer-/storage solution ingredients.

**Fig. 1:**
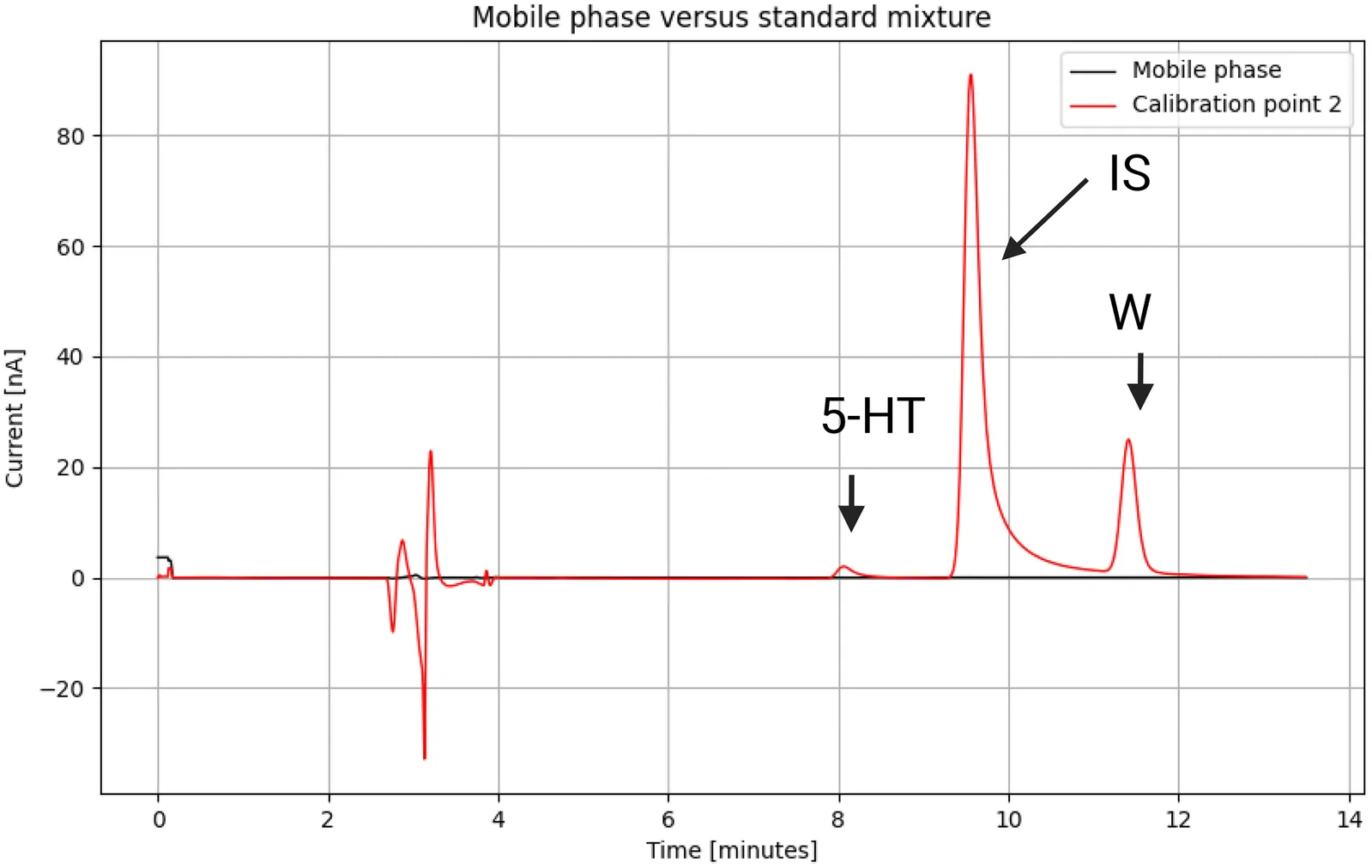
Chromatograms of the standard mixture at calibration point 2 (red line) and the mobile phase (black line) measured at 0.9 V potential of the working electrode against the Ag/AgCl reference electrode. The peaks between 2 and 5 min originate from perchloric acid as part of the standard mixture and the PP buffer ingredient methanol. Arrows mark the retention times of 5-HT at around 8.1 min, of the IS N-methylserotoninoxalate at around 9.6 min and of W at around 11.4 min. No peaks were observed for the mobile phase at these retention times. Retention time depicted in minutes on the x-axis and current in nA on the y-axis.

The linearity and analytical limits of the method were evaluated at four concentration points in a mixed standard solution. The standard samples were prepared as described in Table 1, Table 2. Calibration curves were built by plotting the peak area of the compounds against respective concentrations. Linearity was tested with linear regression using the Clarity software. The limit of detection (LOD) was defined as the concentration of the compounds which generated a signal-to-noise (S/N) ratio of 3 and the limit of quantification (LOQ) was defined as the concentration that generated a signal-to-noise ratio of 10 (see Table 3). The calibration curves for both metabolites are presented in Fig. 2. Quadratic regression showed no differences in the regression coefficient compared to linear regression (R²=0.9997 for 5-HT and R²=0.9998 for W for quadratic and linear regression). For the initial test measurements for the method validation linear regression was applied. During the successive stages of the analysis of the samples from the observational study it became evident that a quadratic fit improved the quality of data, especially of the lower concentrations with calibration being accepted if R² ≥ 0.999 for 5-HT and ≥0.998 for W. The choice of regression model exerted only a minor influence on the group means from the two study cohorts. A comparison of the calculated 5-HT concentrations measured in the serum samples using the different types of regression is shown in **Supplementary Figure 1**.

**Table 3:** Results for the determination of linearity, limits of detection and quantification. Linearity tested with n= 1 in duplicates for all concentration points, LOD calculated with n= 14 and LOQ calculated with n= 11 for all concentration points. The retention time was calculated with n= 11.

Analytes		
	5-HT	W
Regression equation	y = 0.17x+1.1946	y = 0.1435x+19.938
Regression correlation	0.9997	0.9998
LOD (µM; S/N=3.0)	0.0052±0.0017	0.0019±0.0005
LOQ (µM; S/N=10.0)	0.0174±0.0058	0.0065±0.0016
Retention time (min)	8.1±0.1	11.5±0.1

**Fig. 2:**
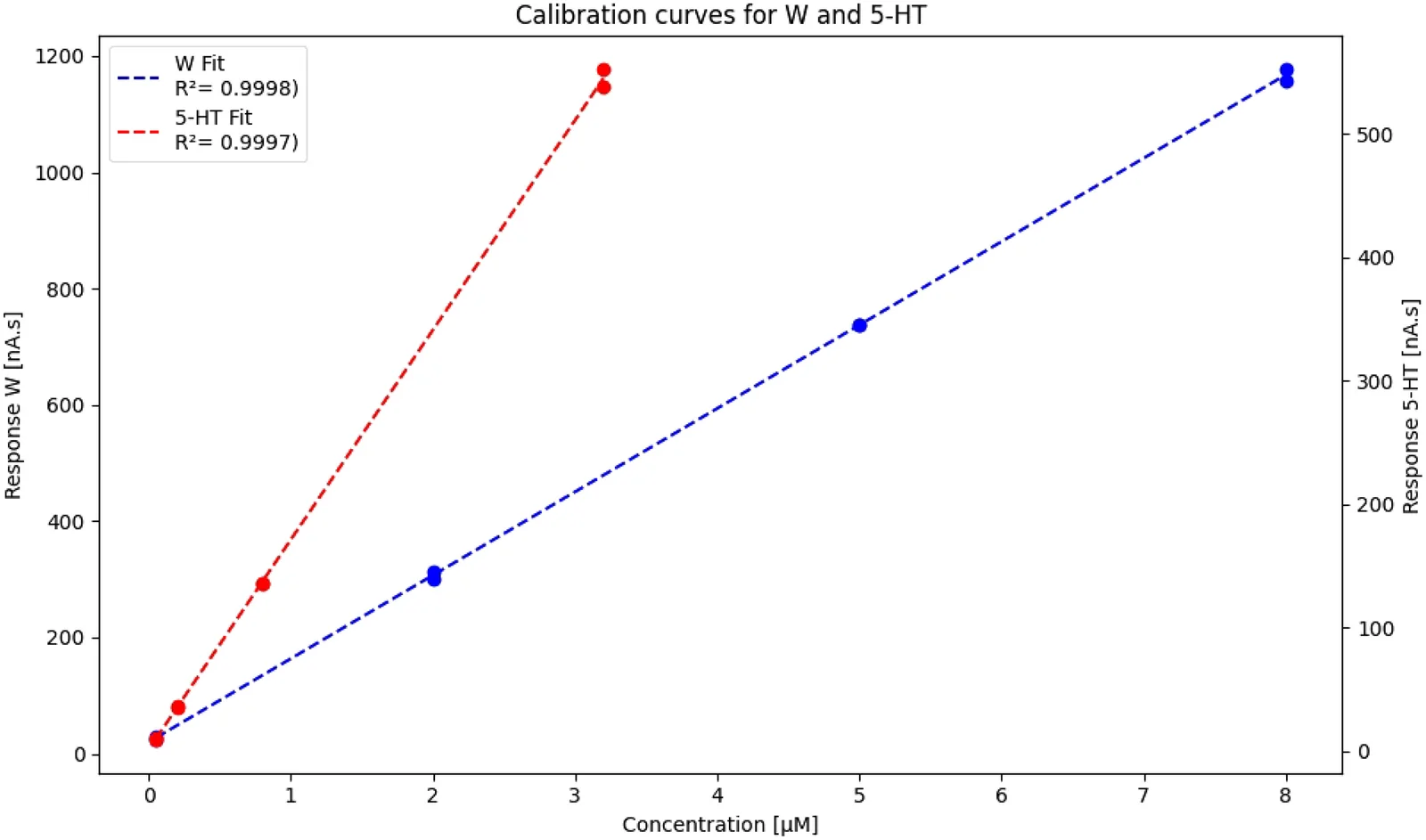
Calibration curves for W and 5-HT using a linear regression model with n=2 for each calibration point. The peak areas of 5-HT on the left y-axis axis and of W on the right y-axis (in nA.s) are plotted against the respective concentrations (for 5-HT: 0.05 µM, 0.2 µM, 0.8 µM and 3.2 µM; for W: 0.05 µM, 2.0 µM, 5.0 µM and 8.0 µM). The regression coefficent for 5-HT is 0.9997 and for W 0.9998.

The intra-day precision and accuracy were evaluated on four different concentration points in a mixed standard solution by repeated measurements. The obtained relative standard deviation (RSD) was based on the concentrations read against the respective concentrations. The inter-day precision was evaluated on the basis of the following standards: 50-3200 nM 5-HT and 50 nM-8 µM W. The calculation was based on n=14 measurements for both 5-HT and W with the exception of the calibration point 50 nM for W (here n=8). Results are provided in Table 4.

**Table 4:** Results for the determination of precision and accuracy. Accuracy and inter-day precision for 5-HT calculated with n=14 for all concentration points, accuracy and inter-day precision for W calculated with n=8 for the concentration point 50 nM (marked with *) and n=14 for all other concentration points.

Analytes	Concentration [µmol/L]	Intra-day precision (RSD, %, n=2)	Inter-day precision (RSD, %, n=14/*8)	Accuracy (mean±SD, n=14/*8)
5-HT	0.05	2.8	9.3	100.9±9.4
	0.2	0.3	3.0	100.4±3.0
	0.8	1.0	0.6	99.9±0.6
	3.2	0.0	0.2	100.0±0.2
W	0.05*	4.8	52.1*	199.1±103.7*
	2.0	2.1	9.0	103.4±9.3
	5.0	1.0	3.3	99.1±3.3
	8.0	0.1	1.0	99.9±1.0

The stability of the two metabolites in the precipitated and diluted samples over a longer period of time has already been addressed in other publications [17] and was therefore not evaluated again. The stability of the standard WS ingredients 5-HT and W was tested by comparing the peak areas after 1 day of storage at 4°C (one sample prepared on day 1 and measured as duplicate on day 1 (n=2) followed by a second measurement of the same sample preparation on day 2 (n=1)), which represents a usual storage time in the auto-sampling unit. The two analytes showed a slight increase of the peak area of approximately 7% (5-HT) and 2% (W) after one day storage at 4°C in the auto-sampling unit. The stability was calculated by comparing the response from the second day with the mean value of the two measurements from the first day.

The recovery was evaluated by measuring unspiked human serum, serum spiked with two 5-HT concentrations and constant W before the precipitation step, and spiked HPLC water (300 nM 5-HT and 10 µM W). The recovery rate was calculated by comparing the measured concentrations of the unspiked serum and spiked serum. The recovery of serum spiked with 300 nM 5-HT (n=8) was 92.66±8.64%, while it was just 85.29±10.35% at the higher spiking level (1000 nM, n=4). In water (n=3) chosen as a blank the recovery was in average 115.9% ±15.2% for 5-HT. The recovery of W was 92.6±13.01% in the samples with 300 nM of spiked 5-HT and 92.83±14.49% in the samples with 1000 nM of spiked 5-HT (in all these samples 10 µM W spiked). In water, 93.3%±2.5% of the W could be recovered. For the unspiked serum n=4.

Data were acquired and analyzed using the Clarity software. Statistical analysis was performed using GraphPad Prism 10.5.0.

## Method optimization

Based on the preceding outcomes, the chromatographic optimized conditions for separating 5-HT and W were identified. The results of the measurements of 5-HT and the IS at three different potential levels used, are presented in Fig. 3. At 0.8 V potential the peak height of 5-HT is approximately 49%, and of the IS approximately 60%, of the peak height at 1.0 V potential. Based on these results, 0.9 V potential was chosen for further measurements, representing a compromise of peak size and noise/drift during the measurement. The electrochemical flow cell (SenCell) allows for a stepless adjustment of the spacing setting and thus an adjustment of the working volume. Reducing the working volume increases the linear flow rate, thereby enhancing sensitivity - while the noise remains nearly unchanged. Switching the spacing setting from position 2 to position 1 corresponds to a 50% reduction in spacing from 50 µm to 25 µm. The adjustment of the cell working volume to the position 1 resulted in increased peak size. This, in turn, simplified the automatic peak detection and integration as shown in Fig. 4.

**Fig. 3:**
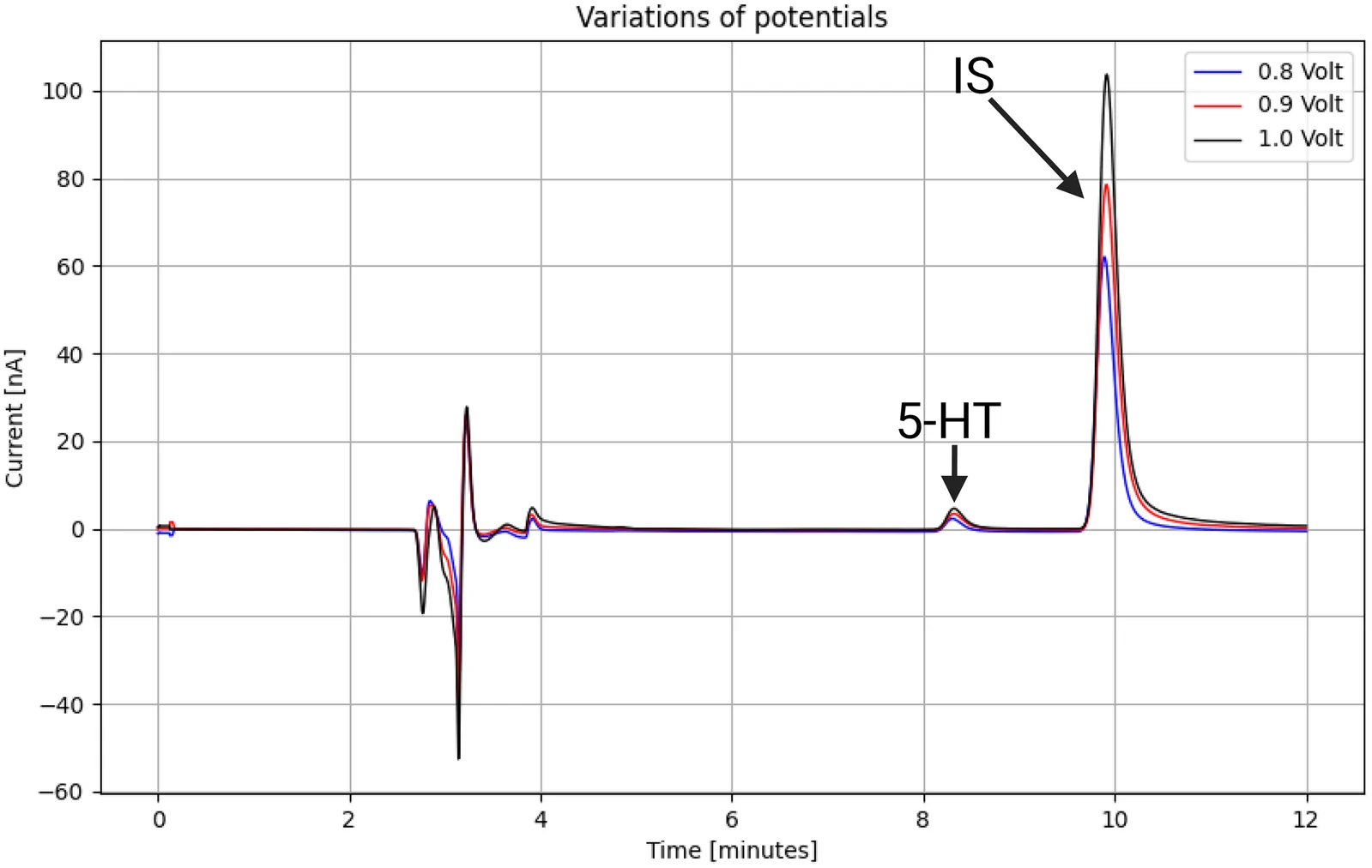
Chromatograms of a standard mixture solution containing 500 nM 5-HT and 8 µM of the IS N-methylserotoninoxalate measured at three potential levels of the working electrode (0.8 V; 0.9 V and 1.0 V in blue, red and black respectively) against the Ag/Ag reference electrode with the 5-HT peaks at around 8.3 min and the IS peaks at around 9.9 min. The retention time in minutes is depicted on the x-axis and the current in nA on the y-axis.

**Fig. 4:**
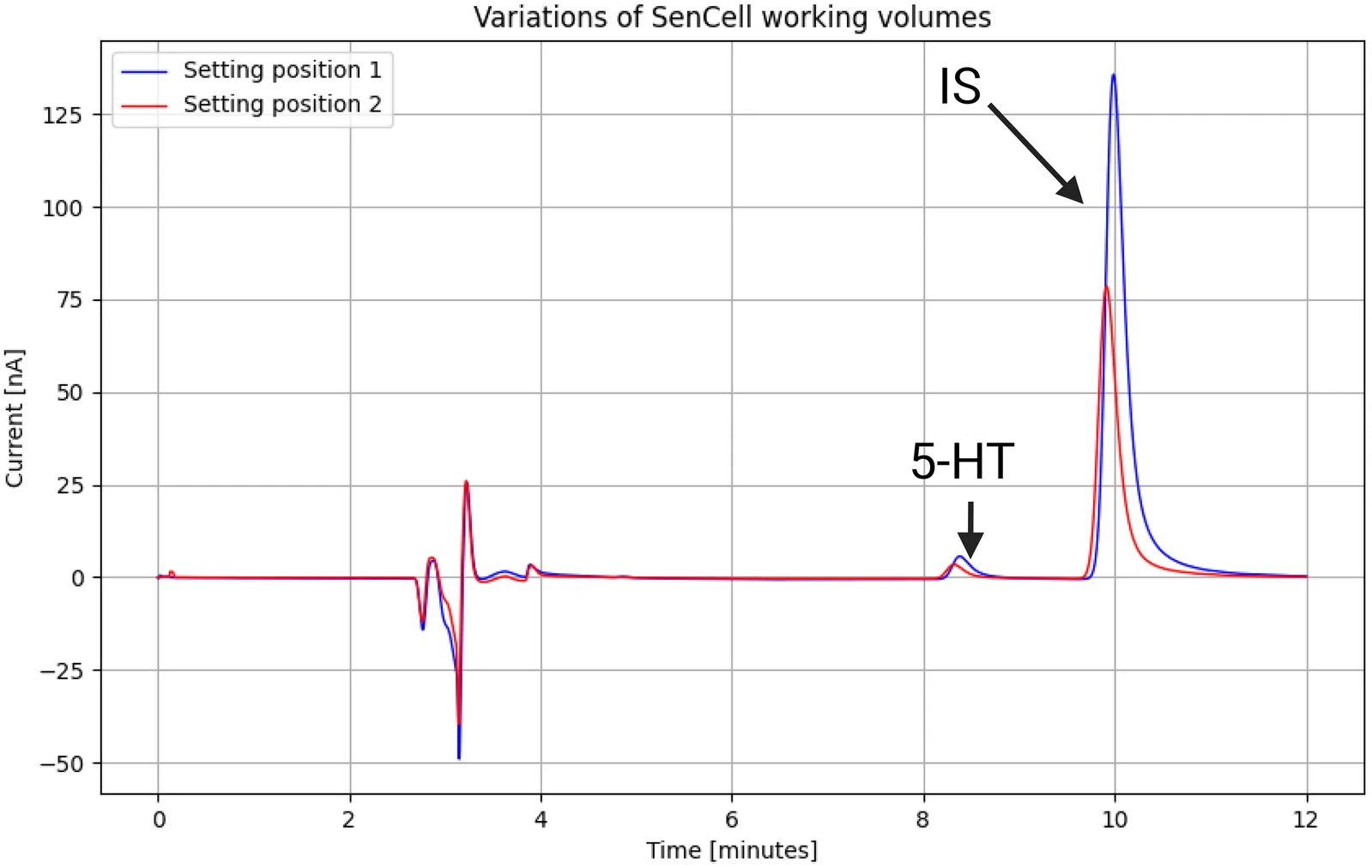
Chromatograms of a standard mixture solution containing 500 nM 5-HT and 8 µM of the IS N-methylserotoninoxalate measured at 0.9 V potential of the working electrode against the Ag/AgCl reference electrode with the 5-HT peaks at around 8.3 min and the IS peaks at around 9.9 min and varied SenCell working volumes represented through varied setting positions (setting position 1 and 2 in blue and red). The retention time in minutes is depicted on the x-axis and the current in nA on the y-axis.

The initial protocol for the serum precipitation started with the addition of 20 µL PP buffer to 10 µL serum followed by the addition of 70 µL of perchloric acid/EDTA to the supernatant. This resulted in sample dependent supernatant volume differences and in the end non-reproducible results. Doubling sample and PP buffer volumes for the precipitation step made it possible to always transfer the same amount of supernatant to each vial to be made up to 100 µL with perchloric acid/EDTA. The addition of IS directly into the mixed standard solutions or into the perchloric acid/EDTA increased the repeatability as measured by the comparability of the IS concentration in the standards and the samples without adding a lot of small volume pipetting steps. Decreasing the standard concentrations of 5-HT after our first human serum sample measurements (initially: 50 nM, 2 µM, 5 µM and 8 µM) to lower ranges helped to improve the coverage of the standard for the expected concentrations in the samples (optimized: 50 nM, 200 nM, 800 nM, 3.2 µM). This made it possible to also detect and analyse 5-HT in samples with lower concentrations (see Fig. 5 representing a human serum sample with 5-HT concentration lower than 500 nM).

**Fig. 5:**
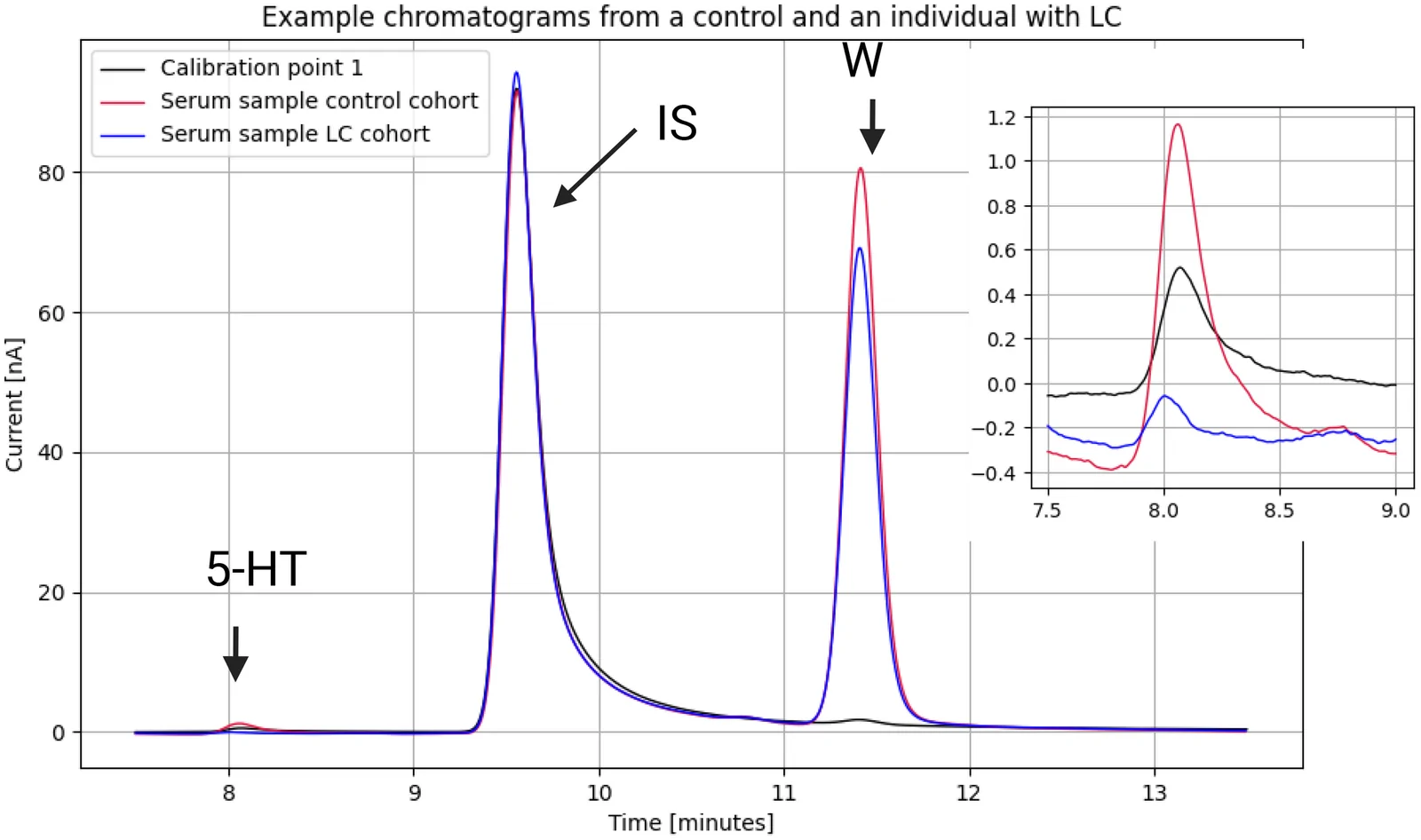
Chromatograms of a standard mixture solution containing 50 nM 5-HT and W and 8 µM of the IS N-methylserotoninoxalate (black line) measured at 0.9 V potential of the working electrode against the Ag/AgCl reference electrode in comparison to a sample from an individual with low 5-HT concentration in serum from the LC cohort (blue line) and a serum sample from an individual from the control cohort (red line). The 5-HT peak is around 8.1 min, the IS peak at 9.6 min and the W peak at 11.4 min. Insert depicts an enlarged view of the 5-HT peaks of the standard mixture solution in comparison to the serum samples with the retention time in minutes on the x-axis and the current in nA on the y-axis.

Using the optimized method, cryo-preserved serum samples from two cohorts (LC cohort and control cohort) were then measured for verification purposes. Serum 5-HT levels in the LC cohort (727.6±274.3nM) were similar to those in the control cohort (815.1±273.4 nM, p=0.30) (see Fig. 6a) and comparable to results in other recently published studies [21,22]. The measured serum W levels showed also no significant difference between the LC cohort (52.68±8.39 µM) and the control cohort (55.32±12.77 µM, p=0.57) (see Fig. 6b). An overview of the basic demographic characteristics of the cohorts can be found in the **Supplementary Table 1.**

**Fig. 6:**
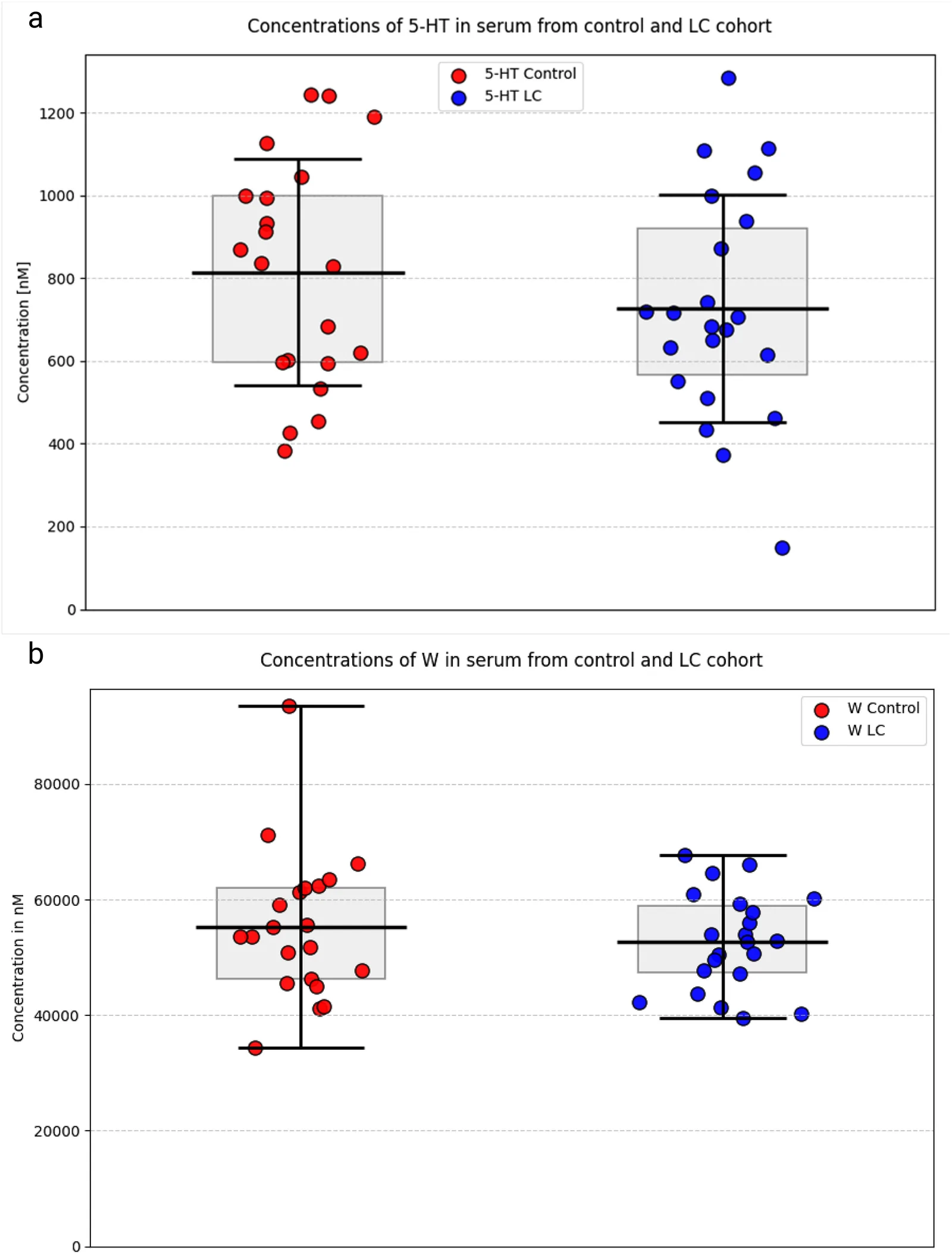
Box plots of 5-HT (a) and W (b) concentrations in serum samples of individuals of both the LC (in blue) and the control cohort (in red). The gray box represents the interquartile range between the third and first quartiles and the horizontal line depicts the mean concentration. The error bars show the standard deviation from the mean concentration.

## Limitations

With the chosen chromatographic conditions, a complete separation of the peaks of W and the IS at concentrations below 2 µM may not always be possible. This becomes particularly apparent when comparing the accuracy values, as the accuracy value for 0.05 µM W is very low compared to the accuracy values of the higher standard concentrations (mean 199% with high RSD). With a final dilution of the samples of 1:10, a measured sample concentration of 0.05 µM W corresponds to a concentration of 0.5 µM W in serum, which is significantly (approximately a factor of 100) below the average for healthy subjects [22]. If the method is to be used in subjects where low W serum concentrations are expected, changing the flow rate or increasing the column length might improve the peak shape/size and finally the resolution. In our study cohorts all measured serum concentrations were higher than 20 µM.

Peaks in serum were identified by comparing retention time and peak shapes with the respective peaks in the standard mixture, which also included the IS. We included 5-HTP (5-Hydroxytryptophan), a direct precursor of 5-HT, in the standard mixture beside 5-HT and W in our first preliminary test runs. The retention time was around 2.8-3.2 min before 5-HT. As only low amounts of 5-HTP were detectable in these serum test samples, further validation was conducted without addition of 5-HTP. However, other potentially electroactive amino acids beside W (e.g. cysteine and tyrosine) were not tested. Therefore, we cannot entirely rule out that some of the peaks of 5-HT and W could be caused by the presence of other amino acids.

## Ethics statements

The study was approved by the Ethics Committee of the Medical Faculty of the Otto-von-Guericke University Magdeburg (registration number 17/22). All procedures involving human participants were in accordance with the ethical standards of the institutional research committee and with the 1964 Helsinki Declaration and its later amendments or comparable ethical standards. Prior to participation, all subjects signed informed consent forms.

## CRediT author statement

**Dorothee Licht:** Methodology, Formal Analysis, Investigation, Writing – Original draft, Visualization. **Alexander Duscha**: Conceptualization, Formal Analysis, Writing – Review& Editing, Supervision. **Tobias Hegelmaier**: Conceptualization. **Aiden Haghikia**: Conceptualization, Writing – Review& Editing, Supervision, Funding Acquisition. **Christiane Desel**: Conceptualization, Formal Analysis, Writing – Review& Editing, Supervision.

## Related research article

None.

## Declaration of competing interest

The authors declare the following financial interests/personal relationships which may be considered as potential competing interests:

This work was funded by the Saxony-Anhalt Excellence Initiative pilot project “Cognitive Vitality”. The funder had no role in the design, data collection, data analysis, and reporting.

## Data Availability

Data will be made available on request.
