# Supplementary material for: Simultaneous determination of serotonin and its precursor tryptophan in small volumes of human serum using HPLC-ECD
Source: MethodsX. 2026 Oct 2;17:104190. doi: 10.1016/j.mex.2026.104190 (PMC13675853; doi:10.1016/j.mex.2026.104190)
Supplement: Supplementary file 1 [file mmc2.pdf]

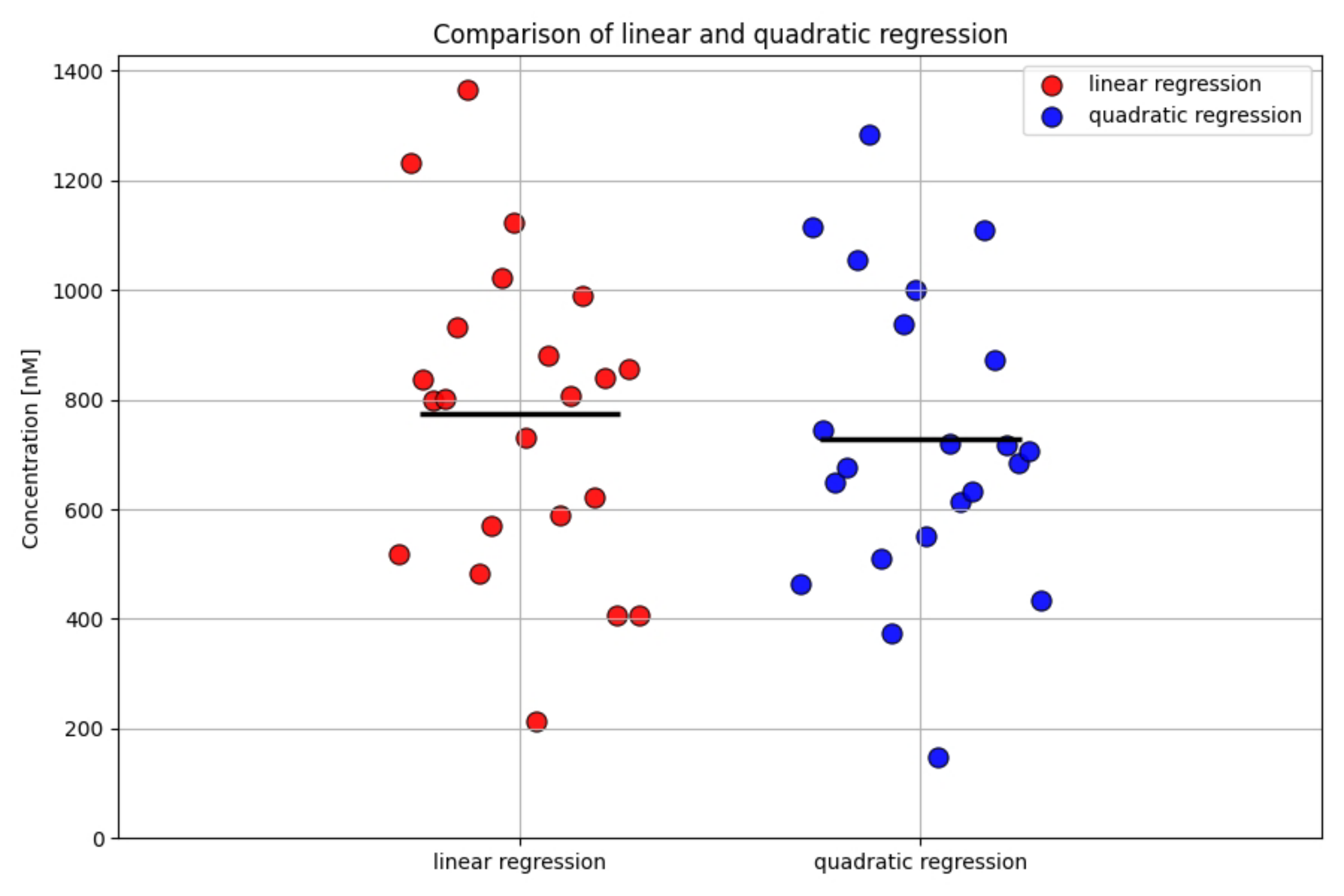

Comparison of linear and quadratic regression
Concentration [nM]
0
200
400
600
800
1000
1200
1400
linear regression
quadratic regression
linear regression
quadratic regression
