# Supplementary material for: Simultaneous determination of serotonin and its precursor tryptophan in small volumes of human serum using HPLC-ECD
Source: MethodsX. 2026 Oct 2;17:104190. doi: 10.1016/j.mex.2026.104190 (PMC13675853; doi:10.1016/j.mex.2026.104190)
Supplement: Supplementary file 2 [file mmc3.pdf]

Universitätsklinik für
Neurologie
Prof. Dr. med. Aiden Haghikia
Direktor
Universitätsklinikum Magdeburg A.ö.R.

Der Einfluss der Darm-Hirn-Achse auf subjektive sowie objektive Parameter der Fatigue bei Patienten mit Multipler Sklerose; ME/CFS und Long-COVID-Syndrom

Studienteilnehmer: ________________Teilnehmer-Nr.: _________

(Name, Vorname)

Geb.-Datum: __________________

Hiermit erkläre ich meine Einwilligung zur Teilnahme an den immunologischen und mikrobiologischen Untersuchungen gemäß der vorstehenden Information. Durch meine Unterschrift bestätige ich, dass ich den Inhalt der Information gelesen und verstanden habe. Ich hatte Gelegenheit, Fragen zu stellen, die mir zufriedenstellend beantwortet wurden. Ich weiß, dass meine Teilnahme freiwillig ist und ich meine Einwilligung jederzeit ohne Angabe von Gründen widerrufen und die Vernichtung meiner Blut-/Stuhl-/ Gewebeproben fordern kann, ohne dass mir daraus irgendwelche Nachteile entstehen.

( ) Ich bin ausdrücklich damit einverstanden, dass die Zwecke wissenschaftlich- medizinischer Forschung, für die meine Probe verwendet wird, heute noch nicht eingegrenzt werden.

( ) Ebenso bin ich damit einverstanden, dass Proben in pseudonymsierter Form an Kooperationspartner für wissenschaftliche Analysen weitergegeben werden dürfen.

Mir ist bewusst, dass ich keinen persönlichen Vorteil aus diesem Forschungsprojekt haben werde und dass mir die Ergebnisse nicht mitgeteilt werden. Nur dann, wenn aus den mir entnommenen Biomaterialien wider Erwarten medizinische Erkenntnisse gewonnen werden, die für die Erhaltung oder Wiederherstellung meiner eigenen Gesundheit von erheblicher Bedeutung sind, wird mein Studienarzt darüber informiert, damit er mit mir das weitere Vorgehen besprechen kann.

Falls gewünscht, bitte ankreuzen:

( ) Ich möchte **nicht**, dass mein Studienarzt über Befunde im vorstehend genannten Sinn informiert wird.

Ich möchte die Verwendung meiner Blut-/Stuhl-/Gewebeproben wie folgt eingrenzen:

....

Universitätsklinik für Neurologie
Prof. Dr. med. Aiden Haghikia
Direktor
Universitätsklinikum Magdeburg A.ö.R.

Eine Kopie dieser Information und Einwilligungserklärung habe ich erhalten. Das Original verbleibt in der Studienstelle.

____
Name des Studienteilnehmers in Druckbuchstaben

____________________ _________________________
Ort, Datum (vom Teilnehmer einzutragen) Unterschrift Teilnehmer

Ich habe das Aufklärungsgespräch geführt und die Einwilligung des Forschungsteilnehmers eingeholt.

____
Name des Studienarztes in Druckbuchstaben

____________________ _________________________
Ort, Datum Unterschrift des Studienarztes
