# Supplementary material for: Simultaneous determination of serotonin and its precursor tryptophan in small volumes of human serum using HPLC-ECD
Source: MethodsX. 2026 Oct 2;17:104190. doi: 10.1016/j.mex.2026.104190 (PMC13675853; doi:10.1016/j.mex.2026.104190)
Supplement: Supplementary file 3 [file mmc1.docx]

**Supplementary Table 1:** Demographic characteristics of the LC cohort and the control cohort and measured serum concentrations of 5-HT and W in both cohorts. The p-value was determined using Mann-Whitney test for the age and W serum concentration, Chi-square test for the gender and Welch´s test for the 5-HT serum concentration.

|  | Cohort LC (n=22) | Controls (n=21) | p-value |
| --- | --- | --- | --- |
| Age, mean±SD | 40.27±12.56 | 32.19±11.60 | 0.04* |
| Female sex, n (%) | 17 (77.27) | 15 (66.67) | 0.44 |
| 5-HT serum concentration in nM±SD | 727.6±274.3 | 815.1±273.4 | 0.30 |
| W serum concentration in nM±SD | 52678±8390 | 55324±12767 | 0.57 |
